# Retraction Note: Multidisciplinary management for Peutz–Jeghers syndrome and prevention of vertical transmission to offspring using preimplantation genetic testing

**DOI:** 10.1186/s13023-023-02949-2

**Published:** 2023-10-11

**Authors:** Xiqiao Xu, Ruifeng Song, Kaiyue Hu, Ya Li, Haixia Jin, Bing Chen, Wenyan Song, Yile Zhang, Jiawei Xu, Yingpu Sun

**Affiliations:** 1 Center for Reproductive Medicine, Henan Key Laboratory of Reproduction and Genetics, The First Affiliated Hospital of Zhengzhou University, 450052 Zhengzhou, Henan China; 2 Henan Key Laboratory of Reproduction and Genetics, The First Affiliated Hospital of Zhengzhou University, Zhengzhou, China; 3 Henan Provincial Obstetrical and Gynecological Diseases (Reproductive Medicine) Clinical Research Center, The First Affiliated Hospital of Zhengzhou University, Zhengzhou, China; 4 Henan Engineering Laboratory of Preimplantation Genetic Diagnosis and Screening, The First Affiliated Hospital of Zhengzhou University, Zhengzhou, China; 5 Department of Gastroenterology, First Affiliated Hospital of Zhengzhou University, Zhengzhou, China

**Retraction Note: Orphanet Journal of Rare Diseases (2022) 17:64**

10.1186/s13023-022-02221-z

The Editor-in-Chief has retracted this article after the authors discovered 14 data calculation errors in Table 1. The authors have been invited to submit a new manuscript for peer review. Kaiyue Hu disagrees with this retraction. None of the other authors has responded to correspondence from the Publisher about this retraction.

